# Factors affecting social accountability of medical schools in the Korean context: exploratory factor and multiple regression analyses

**DOI:** 10.1080/10872981.2022.2054049

**Published:** 2022-03-21

**Authors:** Sangmi T. Lee, Eunbae B. Yang

**Affiliations:** a Department of Medical Education, Yonsei University Wonju College of Medicine, Gangwon-do, South Korea; b Department of Medical Education, Yonsei University College of Medicine, Seoul, South Korea

**Keywords:** reponsibility, stakeholders, social contract, HSP model, Korean medical schools

## Abstract

The concept of social accountability of medical schools is becoming increasingly important worldwide, and numerous frameworks and evaluation tools have been developed. This study examined how global concepts work in a specific context by identifying the factors affecting medical schools’ social accountability performance in the Korean context. A survey was conducted with 40 current deans of medical schools and 15 medical education experts in Korea to assess their opinions on the implementation of social accountability of medical schools. A questionnaire survey comprising five key factors, including 39 items, was developed based on a literature review. Exploratory factors were analyzed to derive factors affecting social accountability Multiple regression analysis was conducted to determine the importance of each factor in the implementation of social accountability of medical schools. The exploratory factor analysis revealed that eight factors in three areas influenced the implementation of social accountability by medical schools. The hardware (H) area included the declaration of social accountability and physicians, organizations and systems for implementing social accountability, and physical environment and finance. The software (S) area included curriculum design-related social accountability and monitoring and evaluation system. The partner (P) area included the proximity between partners, building partnerships among stakeholders, and interactions between partners. Multiple regression analysis revealed that ‘interactions between partners’ had the greatest impact on the implementation of social accountability of medical schools. It is a social accountability implementation model that reflects global principles within the Korean context. The HSP model is significant in that individual medical schools can be used in establishing mandated mechanisms for accreditation. Future studies could adapt this model to study standards and indicators in other contexts.

## Introduction

As life expectancy increases with the development of medical technology, health systems across the globe face the common challenges of aging and lack of access to quality health services [1]. Medical education at medical schools in rural areas worldwide is rapidly developing; however, the simultaneous lack of interest in primary care and the outflow of human resources into metropolitan areas are causing health inequality [2]. The concept of social accountability of medical schools is important to the World Health Organization (WHO) and the World Federation of Medical Education (WFME) [3], who have exhorted changes in the role of health-care workers and advocate designing medical education for them. In addition, the concept of social accountability of medical schools encourages the reform of the health-care system [4]. The Korean government has proposed a new policy on public health care, and the demand for strengthening social accountability education and accreditation of medical schools is increasing.

Since the WHO announced the concept of social accountability in 1995, its implementation in medical schools has been emphasized even further [5]. Medical schools should respond to the demand for social accountability and produce graduates whose professionalism reaches beyond being good practitioners and who serve as agents of change in the health system [6]. To be socially accountable, medical schools should develop a positive impact in the communities they serve, train competent health professionals who can address populations’ needs, and define their health priorities jointly with their community, regional, and national health-care stakeholders [7].

The Alma-Ata Declaration (1978) and Astana Declaration (2018) called for urgent action by all governments, the global health workforce, and the world community to protect ‘health for all’ people globally [8]. In 2010, the Global Consensus for the Social Accountability of Medical Schools (GCSA) established 10 strategic directions to guide medical schools to become socially accountable by redefining their role, reorienting their education, research, and service, and strengthening governance and partnership with other stakeholders. The consensus also stated that all countries should establish accreditation mechanisms for medical schools [9].

Prior studies on the social accountability of medical schools can be largely divided into research related to concepts of social accountability [4,5,10,11], accountability frameworks and performance models [7,12–15], accountability-related programs [16–19], accountability assessment-related indicators [20–25], partnerships with key stakeholders [2,26,27], and so on. Furthermore, based on the factors affecting the implementation of social accountability in medical schools, previous studies can be categorized into those focused on human resource factors, program factors, organizational factors, partnership factors, and environmental factors. These prior studies provide insight into the development and implementation of social accountability. However, most of these studies focus primarily on global principles, while context-specific examination of the social accountability of medical schools is still lacking in extant literature. In addition, attempts and educational reforms to measure social accountability performance in the areas of education, research, and service of medical schools, such as the grid model, CPU model, THEnet framework, AIDER model, ASPIRE criteria, and CARE model, are emerging [14]. However, it is necessary to fully discuss whether models developed within the USA, Canada, and Europe can be applied to specific countries.

This research, therefore, aimed to examine the key factors affecting the social accountability of medical schools in the Korean context using exploratory factor analysis (EFA) and multiple regression analysis. The findings are expected to help establish an essential mechanism for accreditation in the Korean context and effectively establish a continuous improvement plan for the implementation of social accountability. In the Korean context, medical schools have traditionally emphasized quantitative growth and training of medical personnel centered on patient care from a macro perspective, without much focus on the role of social accountability of medical schools to the community, region, or country. The ultimate purpose of this study was to reconsider the role of medical schools in social accountability within the community, region, and country, and to present a model that could strengthen their performance.

## Materials and methods

### Participants

To identify the factors affecting the implementation of social accountability in medical schools, 55 responses, including those of the current deans of all 40 medical schools in Korea, and 15 from medical education experts in Korea, were incorporated in the final report. The dean of a medical school represents the overall education and the implementation of accountability of the medical school, and medical education experts have expertise related to the implementation of social accountability. The group of 15 medical education experts selected for this study comprised people with more than 5 years of medical education and research experience and was divided into three subgroups (Group A: five medical education majors, Group B: five evaluation experts with experience in accreditation, and Group C: five evaluation experts with self-evaluation experience in medical schools).

Full-time faculty related to medical education, and those with more than 5 years of education experience were selected to meet all the selection criteria suggested in this study. The specific criteria for selecting Group A were those who held a doctorate in medical education or pedagogy, and who were currently serving as full-time faculty in the medical education department. Group B’s expert group related to accreditation comprised full-time faculty who were members of the Korean Institute of Medical Education and Evaluation (KIMEE) and belonged to the Committee on Standards. Group C’s self-evaluation committee chairman and executive secretary expert group were selected from each medical school that had accreditation based on ASK2019, the current accreditation standard for Korean medical schools.

All 40 medical schools’ deans and 15 medical education experts selected for the study were assured that they would not be identified through any report in this study and that their data would be anonymized, after which they provided written consent to participate. This study was approved by the Institutional Review Board at Yonsei University Health System (Y-2020-0087).

### Materials

We developed a survey tool to identify the key factors influencing the social accountability performance of medical schools, called the ‘Opinion Survey on the Importance of Factors Influencing the Implementation of Social Accountability by Medical Schools,’ consisting of 39 questions based on five factors. We conducted a literature review to establish a theoretical basis for social accountability concepts and performance in the preliminary phase. The tool was reviewed based on the questions used in previous studies to ensure the suitability of its contents. These were the concept of social accountability of medical schools in the grid model [4], the conceptualization-production-usability (CPU) model [10], and 10 areas of GCSA’s standards [9,23], and an evaluation framework developed by The Training for Health Equity Network (THEnet) [13,28], the student’s toolkit on social accountability in medical schools developed by the International Federation of Medical Student’s Association (IFMSA) [29,30], and the criteria of social accountability area in the A School Programmed for International Recognition of Excellence in Education (ASPIRE) [31] that were translated within the Korean context. Based on previous research, this study hypothesized five key factors (human resource factors, program factors, organization internal factors, partnership factors, and environmental factors), with a total of 39 items, as indicated in Table 1. The questionnaire comprised three sections. The first section included questions related to medical school information. The second section consisted of questions assessing the implementation of social accountability, and the respondents were required to rate their agreement on each statement using a 5-point Likert scale, ranging from 1 = strongly disagree to 5 = strongly agree. The third section related to suggestions.

**Table 1.** The 39 items assessing the five hypothesized factors affecting the implementation of social accountability

Factor	Items assessing factors affecting implementation of social accountability	Reference
1. Human resource (HR)	1.1 Members’ understanding of the concept of social accountability	[7,32]
	1.2 Members’ empathy for the importance of implementing social responsibility	[4]
	1.3 Leaders’ interest in implementation of social accountability of medical school	[10]
	1.4 Participation of stakeholders in the implementation of social accountability (planning, implementing, evaluating)	[8,55]
2. Education program (ER)	2.1 Organization of education curriculum reflecting the social needs of health care in the community	[33,34]
	2.2 Education programs, projects based on communities for the underprivileged in the society	[35,36]
	2.3 Drive research projects on national and regional health care issues and health inequality	[37],
	2.4 Conduct clinical practice on the patient population in the community where the graduates come from	[13,37,38]
	2.5 Encourage faculty and students to participate in community-based service learning	[39,40]
3. Organization internal (OI)	3.1 Statement on social accountability to establish ideology, mission, or educational objectives	[20,21,41]
	3.2 Organization of a committee or dedicated department in charge of performing social accountability	[42]
	3.3 Establishment of an organizational system for the performance of social accountability	[43]
	3.4 Expertise of dedicated personnel responsible for the performance of social accountability	[42,44]
	3.5 Incentives for implementing social accountability (including performance assessment)	[42,44]
	3.6 Policy to select vulnerable or socially disadvantaged students	[42,44]
	3.7 Establish an organizational culture that emphasizes social accountability	[15,45]
	3.8 Encourage students to pursue a career in primary care	[34,46]
	3.9 Budget and financial support for the performance of social accountability	[16]
	3.10 Environment of education, research, and medical facilities related to the performance of social accountability	[20,21]
	3.11 Development of social accountability framework model	[15,25,47]
	3.12 Development of indicators to measure the implementation of social accountability	[20,21,23]
	3.13 Establishment of social accountability monitoring system	[28]
	3.14 Periodic assessment of social accountability performance	[14]
	3.15 Train graduates who influence society’s needs as the main agents of health care system change beyond fostering good clinical doctors	[48,49]
	3.16 Regularly hold report and seminar evaluation activities for performing social accountability	[14,28]
4. Partnership interaction (PS)	4.1 Establish goals with key stakeholders	[11,45]
	4.2 Establish partnerships with diverse stakeholders	[45]
	4.3 Conduct regular monitoring of health care needs in the society	[23]
	4.4 Satisfaction of graduates working at national and regional health care institutions	[34,46]
	4.5 Seamless communication and information exchange with key stakeholders	[3,5]
	4.6 High understanding of key stakeholders	[22]
	4.7 Balance of role-sharing between medical schools, community, government	[9,17]
	4.8 Regularly hold social accountability consortiums with key stakeholders	[30,54]
	4.9 Evaluate whether social accountability performance activities have a positive impact on the society	[4,50]
5. Environment (EV)	5.1 Social accountability standards in accreditation	[22,51]
	5.2 State support for implementation of outstanding social accountability of medical school	[9,17]
	5.3 Possibility of access to targets that carry out social accountability	[7,10,52]
	5.4 Ease of establishing organic cooperative relationships with key stakeholders	[3,8],
	5.5 Association of health care needs and priorities in medical school and community	[53]

### Data collection and analysis

The survey was conducted from 24 July to 7 September 2020, and all 40 medical schools’ deans and 15 medical education experts responded to the survey (100% response rate). A reliability analysis of the questionnaire indicated that skewness ranged from −.41 to .11 and kurtosis from −.95 to −.38, meeting the univariate normality assumptions, with the absolute values of skewness and kurtosis not exceeding 2 and 7, respectively. Therefore, it was considered appropriate to verify the significance of the study model using the maximum likelihood method, as it satisfies the assumption of normal distribution for all variables. An exploratory factor analysis was conducted based on 37 questions, with the exception of two less question-in-quantity consistencies, by examining the question-in-quantity consistency of question 39.

Exploratory factor analysis was conducted using the maximum likelihood method and direct oblimin rotation method, and Cronbach’s alpha coefficient was calculated to confirm the internal consistency of the questionnaire. The number of factors was determined by considering eigenvalues and scree tests, where the minimum eigenvalue was 1 and the slope changed rapidly in the scree chart. The criteria for interpreting the factors in the final structure were set at .40. The factors used a root-mean-square error of approximation (RMSEA) goodness-of-fit because of its advantage of being less sensitive to sample size and the simplicity of the model; an acceptable goodness-of-fit was determined as a RMSEA value of less than or equal to 10. An exploratory factor analysis was performed using the SPSS statistical package (SPSS for Windows, version 25.0, SPSS Inc., Chicago, IL, USA).

Furthermore, Pearson’s correlation analysis was conducted to compare the magnitude of influence among factors affecting the performance of social accountability at medical schools, and multiple regression analysis was conducted to examine the importance of influence on the performance of social accountability at medical schools. Finally, an analysis of differences between groups was conducted to identify the important factors influencing the social accountability performance of the deans and professional groups of medical schools.

## Results

### Exploratory factor analysis: hardware-software-partner model (HSP model)

An exploratory factor analysis was conducted to determine whether the factors affecting the implementation of social accountability of medical schools were represented by the five factors examined in this study. In the exploratory factor analysis, eight factors with eigenvalues higher than Kaiser’s criterion of 1 were calculated using the maximum likelihood and direct oblimin rotation methods. As illustrated in Table 2, the eight factors accounted for approximately 76.7% of the variance. One item (Item 1.3) with less than 0.40 in common was removed. Four items (Items 1.2, 3.8, 5.2, and 5.5) had factor loadings of less than 0.40. Three items (1.4, 2.5, and 3.5) did not have adequate internal consistency and were excluded. Cronbach’s α for the 29-item HSP Model was 0.96, whereas Cronbach’s α for the eight factors/subscales ranged from 0.71 to 0.95. The correlations between the items were sufficiently large to conduct factor analysis. A decision was made to adopt the 29 items and eight factors of the HSP model for the purpose of validating a framework for implementing social accountability at medical schools in the Korean context. Table 3 provides a summary of the exploratory factor analysis results for the 29-item HSP model (n55). It was named the HSP model in the same sense as structural approaches such as computer hardware, software that actually works, and partnerships with major stakeholders.

**Table 2.** Results of the exploratory factor analysis: HSP model

Item no	Statement	Factor							
		1	2	3	4	5	6	7	8
4–1	Establishment of goals with key stakeholders	.973							
4–2	Establishment of partnerships with diverse stakeholders	.845							
3–12	Development of indicators to measure the implementation of social accountability		.950						
3–13	Establishment of social accountability monitoring system		.934						
3–14	Periodic assessment of social accountability performance		.886						
3–3	Establishment of an organizational system for the performance of social accountability			.930					
3–4	Expertise of dedicated personnel responsible for the performance of social accountability			.858					
3–2	Organization of a committee or dedicated department in charge of performing social accountability			.801					
3–7	Establishment of an organizational culture that emphasizes social accountability			.652					
3–16	Regular holding of report and seminar evaluating activities for performing social accountability			.614					
2–1	Organization of education curriculum reflecting the social needs of health care in the community				.855				
2–2	Education programs, projects based on communities for the underprivileged in the society				.700				
2–4	Conduct clinical practice on the patient population in the community where the graduates come from				.674				
2–3	Drive research projects on national and regional health care issues and health inequality				.699				
1–1	Members’ understanding of the concept of social accountability				.619				
4–6	High understanding of key stakeholders					.882			
4–4	Satisfaction of graduates working at national and regional healthcare institutions					.787			
4–5	Seamless communication and information exchange with key stakeholders					.720			
4–8	Regular holding of social accountability consortiums with key stakeholders					.727			
4–3	Conduct regular monitoring of health care needs in the society					.694			
4–9	Evaluation of whether social accountability performance activities have a positive impact on the society					.692			
4–7	Balance of role-sharing among medical schools, community, government					.636			
5–3	Possibility of access to targets that carry out social accountability						−.708		
5–4	Ease of establishing organic cooperative relationships with key stakeholders						−.531		
3–9	Budget and financial support for the performance of social accountability							.798	
3–10	Environment of education, research, and medical facilities related to the performance of social accountability							.761	
3–11	Development of social accountability framework model							.696	
3–15	Training of graduates who influence society’s needs as the main agents of health care system change beyond fostering good clinical doctors								−.802
3–1	Statement on social accountability to establish ideology, mission, or educational objectives								−.563
Eigenvalues		15.62	2.72	2.19	1.99	1.89	1.59	1.23	1.15
% of variance		42.21	7.36	5.93	5.37	5.11	4.29	3.32	3.10
Total variance		42.21	49.57	55.50	60.87	65.98	70.27	73.59	76.69

F1 = building partnerships among stakeholders, f2 = monitoring and evaluation system, f3 = organizations and system for implementing social accountability, f4 = curriculum design based on social accountability, f5 = interactions between partners, f6 = contributing to proximity between partners, f7 = the physical and financial environment, f8 = the declaration of social accountability and physician workforce.

**Table 3.** Internal consistency of the HSP model

Area	Factor	Subscale	No. of items	Item no.	Cronbach’s alpha coefficient
H (Hardware)	F3	Organizations and system for implementing social accountability	5	3–3, 3–4, 3–2, 3–7, 3–16	0.90
	F7	The physical and financial environmental	3	3–9, 3–10, 3–11	0.85
	F8	The declaration of social accountability and physician workforce	2	5–15, 3–1	0.71
S (Software)	F4	The curriculum design based on social accountability	5	2–1, 2–2, 2–4, 2–3, 1–1	0.84
	F2	The monitoring and evaluation system	3	3–12, 3–13, 3–14	0.95
P (Partner)	F1	Building partnerships among stakeholders	2	4–1, 4–2	0.93
	F5	Interactions between partners	7	4–6, 4–4, 4–5, 4–8, 4–3, 4–9, 4–7	0.92
	F6	Contributing to proximity between partners	2	5–3, 5–4	0.89
			29		0.96

Based on the HSP model, eight factors in three areas influenced the implementation of social accountability by medical schools in the Korean context. The hardware (H) area included the declaration of social accountability and physician workforce, organizations and systems for implementing social accountability, and physical and financial environmental factors. The software (S) area included the curriculum design based on social accountability and the monitoring and evaluation system factors. Finally, the partner (P) area included the proximity between partners, building partnerships among stakeholders, and interactions between partner factors.

A scree plot is a useful method to determine the number of factors that should be retained in an analysis. An examination of the scree plot in the current study, which indicated that the line began to flatten at component number 9, justified the retention of these eight factors. The results of the cross-comparisons of the 2-factor, 3-factor, 4-factor, 5-factor, 6-factor, 7-factor, and 8-factor models are shown in Table 4 to evaluate the suitability of the 8-factor models identified in this study.

**Table 4.** Goodness-of-fit indices: root-mean-square error of approximation (RMSEA)

Factor model	KMO	X^2^	df	p	RMSEA	Described variance values
2	.71	1002.98	593	.000	0.11	49.57
3	.71	893.92	558	.000	0.11	55.50
4	.71	783.84	524	.000	0.10	60.87
5	.71	699.72	491	.000	0.09	65.97
6	.71	627.16	459	.000	0.08	70.26
7	.71	568.58	428	.000	0.08	73.58
8	.71	502.48	398	.000	0.07	76.68

Cut-offs used to indicate goodness of fit: RMSEA ≤0.08

### Key influencing factor and differences in perceptions between groups

Pearson’s correlation analysis between factors affecting social accountability is shown in Table 5, where all factors are represented as static to important, which indicates increasing importance as factors increase.

**Table 5.** Pearson’s correlations of variables

Factor	Importance
The declaration of social accountability and physician workforce	^.661**^
The curriculum design based on social accountability	.727**
Organizations and system for implementing social accountability	.823**
The monitoring and evaluation system	.766
The physical and financial environmental	.723**
Contributing to proximity between partners	.778**
Building partnerships among stakeholders	.772**
Interactions between partners	.880**

**p < 0.01

Table 6 indicates the results of a stepwise multiple regression analysis of the effect size of the eight factors that affect the implementation of social accountability of medical schools. Among these, ‘interactions between partners’ was the most salient factor in the implementation of social accountability of medical schools in the Korean context. Excluding the partnership building factor, seven factors demonstrated approximately 97.8% (*R*^2^ = .978) explanatory power (model fit F = 303.642, p < .001). Following ‘interactions between partners’ (*ß *= .292), being the most influential factor, ‘the curriculum design based on social accountability’ (*ß *= .261), ‘organizations and system for implementing social accountability’ (*ß *= .210), ‘physical and financial environment’ (*ß *= .163), ‘monitoring and evaluation’ (*ß *= .131), ‘the declaration of social accountability and physician workforce’ (*ß *= .120), and ‘contributing to proximity between partners’ (=.090) contributed, in this order, to the implementation of social accountability of medical schools in Korea.

**Table 6.** Multiple regression analysis of the HSP model

Variable	Unstandardized coefficient		Standardized coefficient	*t*	VIF	R^2^	F
	B	SE	*ß*				
(Constants)	.127	.088		1.434		0.978	303.642***
Interactions between partners	.230	.026	.292	8.814**	2.615		
The curriculum design based on social accountability	.228	.025	.261	9.111***	1.960		
Organizations and system for implementing social accountability	.144	.020	.210	7.092***	2.081		
Physical and financial environment	.115	.020	.163	5.640***	2.002		
Monitoring and evaluation	.092	.021	.131	4.314***	2.193		
The declaration of social accountability and physician workforce	.095	.021	.120	4.421***	1.756		
Contributing to proximity between partners	.062	.022	.090	2.777***	2.478		

**p < 0.05 ***p < .001

Table 7 illustrates the results of the independent samples t-test to examine the difference between the deans and experts in their recognition of the importance of the factors influencing the implementation of social accountability of medical schools. The differences between the two groups demonstrated that the deans group scored higher across all factors. They recognized ‘the declaration of social accountability and physician workforce,’ ‘the curriculum design based on social accountability,’ and ‘organizations and system for implementing of social accountability’ as the most important factors.

**Table 7.** t-Test showing differences between deans and experts in evaluation of factors

Variable	Deans (N = 40)		Medical Education Experts (N = 15)		t	p
	M	SD	M	SD		
The declaration of social accountability and physician workforce	4.56	0.47	3.83	0.52	4.974***	0.000
The curriculum design based on social accountability	4.27	0.58	3.97	0.38	2.259*	0.030
Organizations and system for implementing social accountability	3.96	0.62	3.45	0.68	2.605*	0.012
Monitoring and evaluation	4.02	0.65	3.98	0.66	0.906	0.369
Physical and financial environment	4.06	0.73	4.02	0.66	0.963	0.341
Contributing to proximity between partners	4.10	0.67	3.80	0.62	1.504	0.138
Building partnerships among stakeholders	4.18	0.67	3.80	0.65	1.873	0.067
Interactions between partners	4.08	0.60	3.68	0.43	2.400*	0.020
Total	4.13	0.50	3.76	0.34	3.055**	0.004

*p < .05, **p < .01, ***p < .001

### Barriers and challenges in the implementation of social accountability of medical schools

**Table 8 describes the factors that hinder the implementation of social accountability of medical schools and action strategies in hardware, software, and partner areas.**

**Table 8.** Barriers and challenges of the HSP model

Area	Hindrance factor	Action strategy
H (Hardware)	Unbalanced regional distribution of incoming students Lack of dedicated organization and staff within the medical school Lack of financial support and physical conditions	Equal opportunity and priority to students from underrepresented communities Commission dedicated to social accountability (department) and personnel dedicated to social accountability Support for financial projects for the implementation of social accountability of medical schools, hospitals, communities, and government agencies
S (Software)	Absence of social accountability implementation model reflecting the characteristics of medical schools Lack of link between community and medical school curriculum Lack of programs for post graduates	Development of social accountability implementation model reflecting the characteristics of medical schools Opening and expanding training for community-linked services learning Development of training programs reflecting the context of graduates
P (Partner)	Poor understanding of the social accountability of partner institutions Unclear or weak governance structure between partner organizations Lack of shared vision among stakeholders	Establishing and sharing social accountability concepts with key stakeholders Continuous interchange and cooperation between medical schools and communities Joint consortium of medical schools, hospitals, and government agencies

## Discussion

The results of this study support the outcome of previous studies that indicated that recognition and performance of the concept of social accountability of medical schools can vary depending on the national and social context of each medical school. The impact factors of social accountability performance could be explained using the HSP model consisting of eight factors, including building partnerships among stakeholders, interactions between partners, and curriculum design-related social accountability. However, it was also found that some factors, considered important globally, are not a part of the HSP model in the Korean context due to societal and cultural differences. Therefore, the results of this study are consistent with previous findings indicating that the outlook of social accountability differs based on each country’s social and cultural context [3,4,6,12,25].

The HSP model reflects global principles, such as responding to society’s priority health needs, partnership with key stakeholders in the health sector, and establishing mandated mechanisms for accreditation. The model had to be tailored to fit the societal needs in the Korean context, such as policies to select vulnerable people or socially disadvantaged people in Korea, and factors that encourage and support graduates to work in vulnerable areas or primary health care, which are not fully reflected in the global HSP model. These particular needs are related to the social, economic, and political factors that influence the Korean medical field. These factors also have a direct influence on the outlook of medical students in Korea. This is because, unlike advanced countries, such as the USA, Canada, and the UK, where public funds are provided to support medical students during their academic career, medical students in Korea go to medical school immediately after graduating from high school, and the financial burden for their studies is carried by the students and their families. Furthermore, it was found that, in Korea, it is more financially beneficial to work in metropolitan areas. This leads to many graduates seeking employment in a metropolitan setting rather than pursuing careers in health care for the vulnerable or socially disadvantaged. Finally, although 63% of Korean medical schools are located in non-metropolitan areas, a large number of hospitals, local patients, medical personnel, and medical institutions are concentrated in Seoul and the metropolitan area because of the development of transportation such as high-speed railways and IT technology being centered in these areas [54].

There remain many obstacles that must be overcome and practical tasks that must be accomplished to strengthen and promote the social accountability of medical schools within the Korean context. The main barrier is the lack of conceptual awareness among key stakeholders. It may also be that, although schools were accredited before 2014, it was only thereafter that the accreditation standards included components of social accountability. In addition, the Korea Institute of Medical Education and Evaluation was awarded recognition status by the WFME in 2016, which likely also increased medical schools’ interest in understanding social accountability and enforcing accountability standards [54]. Consequently, consensus among key stakeholders had not been reached, resulting in a lack of understanding [55]. This lack of understanding led to a lack of educational programs related to the last step in the process of social accountability.

Prior studies have highlighted the importance of medical schools in establishing a shared vision and social accountability strategies with key stakeholders within the national health-care system to achieve social accountability [2,3,8]. However, it has also been found that this can lead to conflicts among stakeholders [3,5,8]. Therefore, to minimize the resistance of key stakeholders, it is important to build consensus on the social accountability of medical schools worldwide and to establish a clear and improved understanding of these concepts among them. In addition, it is necessary to develop guidelines on social accountability standards that can create a common vision and awareness by holding accountability consortiums with key stakeholders, such as medical schools, countries, and regions, to strengthen and spread the social accountability of medical schools. Partnerships with key stakeholders related to social accountability are very important for medical schools to meet levels of responsiveness and accountability beyond social responsibility.

Consortiums with key stakeholders may include an intercollege consortium that can establish consensus on the concept of social accountability of medical schools, develop joint programs for community, regional, and national levels of social accountability, and share best practices for medical schools. Medical schools and communities can enhance the survey of health and medical challenges in the community, expand the implementation of learning communities and service learning curriculums through establishing relationships with the community, and facilitate continuous interaction through mentoring and tutoring. Finally, medical schools and national government agencies can raise policy interest by developing and promoting standards for implementing social accountability for health care, supporting national policy considerations and budgets, expanding joint research projects at the national level, and holding regular consortiums with major stakeholders [22].

Finally, continuous reflection is needed on how global concepts and accreditation work in various national, social, and cultural contexts. Research on reasoning and reviewing the priorities of social accountability performance in the global and specific contexts is necessary [9,12]. In addition, a comparative study on social accountability between countries is needed. Korea has been verifying social accountability based on WFME’s accreditation standards since 2019, which requires post-monitoring.

A limitation of this study is that it is a study of social accountability attitudes of deans and experts but not social accountability measures of social accountability actions and outcomes in medical schools. Also, it does not examine the perception of social accountability from the perspectives of students and professors. It is also necessary to examine the role of social accountability of medical schools expected by communities, regions, and countries. Finally, future research is needed to investigate the strategies and methods used to carry out social accountability according to the specific situation of each medical school.

## Conclusion

Factors affecting the implementation of social accountability in medical schools are explained as common factors and distinguished factors according to the social and cultural contexts of various countries. The HSP model reflects the global principles of the social accountability of medical schools and can be used by individual medical schools as a checklist for the development of social accountability implementation frameworks. Individual medical schools can strengthen social accountability and establish specific strategies depending on their particular situation and characteristics. A shared perception and partnership among stakeholders are important to strengthen the implementation of social accountability in medical schools. The HSP model presented in this study is novel in that it examines the implementation of social accountability of medical schools in the Korean context. In future studies, the development of standards and indicators to assess the implementation of social accountability of medical schools in different contexts should be pursued.

## Data Availability

The data that support the findings of this study are available on request from the corresponding author, [EBY, nara@yuhs.ac].
